# High amount of fertility reducing tumors and procedures, but no evidence for premature ovarian failure in female Lynch syndrome patients

**DOI:** 10.1007/s10689-024-00357-4

**Published:** 2024-01-27

**Authors:** Sabine Biermann, Michael Knapp, Peter Wieacker, Stefan Aretz, Verena Steinke-Lange

**Affiliations:** 1 Institute of Human Genetics, Medical Faculty, University of Bonn, Bonn, Germany; 2 Institut für Medizinische Biometrie, Informatik und Epidemiologie (IMBIE), University of Bonn, Bonn, Germany; 3 Institut für Humangenetik, Universität Münster, Münster, Germany; 4 National Center for Hereditary Tumor Syndromes, University Hospital Bonn, Bonn, Germany; 5 European Reference Network for Genetic Tumor Risk Syndromes (ERN Genturis), Nijmegen, Netherlands; 6 MGZ – Medical Genetics Center, Munich, Germany; 7 Arbeitsgruppe erbliche gastrointestinale Tumore, Medizinische Klinik und Poliklinik IV – Campus Innenstadt, Klinikum der Universität München, Munich, Germany; 8 Institut für Humangenetik Biomedizinisches Zentrum, Venusberg-Campus 1, Bonn, 53127 Germany

**Keywords:** Menopause, Mismatch-repair genes, Hereditary CRC, Cancer predisposition, Lynch syndrome

## Abstract

Lynch syndrome (LS; HNPCC) patients carry heterozygous pathogenic germline variants in mismatch repair (MMR) genes, which have also been shown to play an important role in meiosis. Therefore, it was hypothesized, that LS might be associated with a higher risk for premature ovarian failure (POF) or earlier menopause. Data on medical gynaecological history, cancer diagnoses and therapy were collected from 167 female LS patients and compared to a population-based control cohort. There was no difference between the age of menopause in patients compared to controls and no evidence for a higher risk of POF in LS patients. However, around one third (35%) of the probands have already had premenopausal cancer and mostly cancer-related treatment affecting fertility before the age of 45 years. Therefore, childbearing time might still be limited in these patients, especially due to the premenopausal cancer risk. LS patients should be informed in time about the elevated premenopausal cancer risks and the possible impact on family planning. This is particularly relevant since the average childbearing age has increased during the last decades.

## Introduction

Premature ovarian failure (POF), also known as Primary ovarian insufficiency (POI), is defined as menopause before the age of 40 years and affects about 1 in 100 women in Germany [1]. The etiology of POF is very heterogeneous including external factors like chemotherapy, radiation, or surgery, as well as internal factors like autoimmune diseases. Additionally, POF can occur in the context of hereditary diseases. Especially germline mutations in the *FMR1*, *FOXL2*, or *BMP15* genes have been identified as monogenic causes of POF by compromising folliculogenesis. However, most cases remain unexplained [1]. 

DNA mismatch repair (MMR) proteins, especially MLH1, are known to play an important role in meiosis, an important part of folliculogenesis, as they are responsible for DNA double strand repair in the context of crossing over, which could be proven in mouse models [1, 2]. In humans, heterozygous pathogenic germline variants in four MMR genes (*MLH1*, *MSH2*, *MSH6*, and *PMS2*) or heterozygous germline deletions of the *EPCAM* gene are causative for Lynch syndrome (LS; also known as hereditary non-polyposis colorectal cancer, HNPCC). LS is a hereditary tumor disposition syndrome, characterized by a high lifetime risk for colorectal cancer, and a broad spectrum of additional tumors including endometrial and ovarian cancer [3]. As MMR genes are involved in meiosis and can cause infertility in mice, we suspected that female LS patients might be prone to POF as well. In addition, the high tumor risk for premenopausal gynaecological cancers amongst others might affect fertility and childbearing in these women as well.

As the knowledge of a reduced fertility would be important for family planning, the aim of this study was to find out if LS patients generally have a lower age at menopause compared to the general population and to identify additional factors impairing fertility in these patients.

## Patients and methods

### Patients

Female patients with genetically confirmed LS were recruited at the Institute of Human Genetics, University Hospital Bonn, one of the centers of the German Consortium Familial Colorectal Cancer as described before [4]. Clinical data of the study participants are collected prospectively and entered into the consortium registry. The study was approved by the ethics committee of the University Hospital Bonn (no. 115/09). All patients provided written informed consent.

241 female LS patients, registered at the study center in Bonn, who were 40 years or older at that time, were contacted by mail and asked, if they were willing to participate in this project. All patients carried a pathogenic germline variant in either *MLH1*, *MSH2*, *MSH6*, *PMS2*, or *EPCAM*. 19 patients refused to participate, 3 had died already, and 52 patients were lost to follow-up. Therefore, in total 167 patients were available for a telephone interview regarding their medical gynaecological history. The interview was carried out following a structured questionnaire, including questions on age at menarche and (if applicable) menopause, pregnancies, hormone intake, gynaecological operations, cancer diagnoses and therapy.

### Controls

Patients age at menopause was compared to a population-based control cohort, that had been recruited by the Robert-Koch-Institut in Germany in the context of the study “DEGS - Studie zur Gesundheit Erwachsener in Deutschland”. The process of data collection within this study has been described before [5]. The dataset provided by the Robert-Koch-Institut included data of 4,283 women, of whom 3,675 also had information on the age at menopause. These women were used as control cohort.

### Statistical analysis

Survival curves for age at menopause were estimated by Kaplan-Meier method and compared between cases and controls by means of the log-rank test. Individuals were treated as censored observations once they had undergone chemotherapy, ovariectomy or hysterectomy. As the main childbearing time is before the age of 45 years, we decided to use this age as threshold for most analyses.

## Results

Of the 167 LS patients, 77 (46.1%) carried a pathogenic variant in *MLH1*, 63 (37.7%) in *MSH2*, 18 in *MSH6* (10.8%), 4 in *PMS2* (2.4%), and 5 patients (3.0%) carried a pathogenic deletion of the *EPCAM* gene. At the time of data collection, the patients were on average 56 years of age (range 40 to 86 years). Age at menarche was 13.3 years on average (median 13 years, range 10.5 to 18 years), which is in accordance with the German general population. Basic clinical and genetic characteristics are summarized in Table 1.

**Table 1:** Description of patient cohort (*n* = 167)

	Number of patients or years	Range or percentage of all study patients (*n* = 167)
Average age at study inclusion (years)	56 y	40–86 y
Average age at menarche (years)	13.3 y	10.5–18
Average number of children	1.9	0–9
Average age at first birth (years)	25.9 y	15–40 y
Average age at first cancer (years)	43.3 y	24–67 y
Any cancer	128	76.7%
Colorectal cancer	109	65.3%
Endometrial cancer	37	22.2%
Ovarian cancer	11	6.6%
Premenopausal first cancer	110	65.9%
Premenopausal hysterectomy	77	46.1%
Premenopausal chemotherapy	40	23.9%

All in all, 128 of the patients (76.7%) had been diagnosed with cancer at the time of study inclusion; about half of these (63 patients; 49.2%) had more than one tumor (up to 5 primary cancers). 48 patients of the cancer patients (37.5%) underwent chemotherapy for at least one cancer, 40 patients (31.3%) were premenopausal at the time of first chemotherapy. Most cancer patients (102 patients; 79.7%) got their first tumor diagnosis before the age of 50 years, 110 women (85.9%) were premenopausal at the time of their first cancer diagnosis.

Medical gynaecological procedures were common in our patient cohort, mainly due to the high cancer risk. Hysterectomy (HE) had been conducted in 92 patients (55.1%), half of them in the context of cancer therapy (51 patients; 55.4%): 37 patients had a diagnosis of endometrial cancer (three with additional ovarian cancer), six had ovarian cancer, and four cervical cancers (or precursor lesions); four patients underwent gynaecological surgery for metastatic colorectal cancer and breast cancer. In 22 patients, HE was conducted due to other medical reasons like uterine myoma or hypermenorrhea. Furthermore,19 patients had opted for prophylactic HE due to the high cancer risk in the context of LS.

Of the 167 study patients, 122 (73.1%) were postmenopausal when the interview was conducted, 50 of these patients (41.0%) had not undergone cancer therapy or gynaecological surgery until then. Age at menopause in these 122 patients ranged between 32 and 55 years. Eight patients (6.6%) were younger than 40 years (between 32 and 39 years) at time of menopause, two in the context of chemotherapy and six due to premenopausal HE and ovariectomy. Another 10 patients were younger than 45 years at time of menopause, six of them also because of premenopausal HE and ovariectomy, four of them had menopausal symptoms after premenopausal HE. In another five patients, menopause occurred naturally without obvious iatrogenic influences of any kind before the age of 45 years. In total, 23 patients were postmenopausal under the age of 45 years (18.9%). Menopause was usually diagnosed by symptoms; measurement of hormone levels in this context had only been carried out in a minority of patients.

We had information regarding childbearing from 159 patients. Of these, 21 women (13.2%) did not have any children at time of study inclusion. Of the 138 patients with children, 103 (74.6%) were younger than 30 years at the time of birth of their first child with an average age of 26 years.

**Fig. 1:**
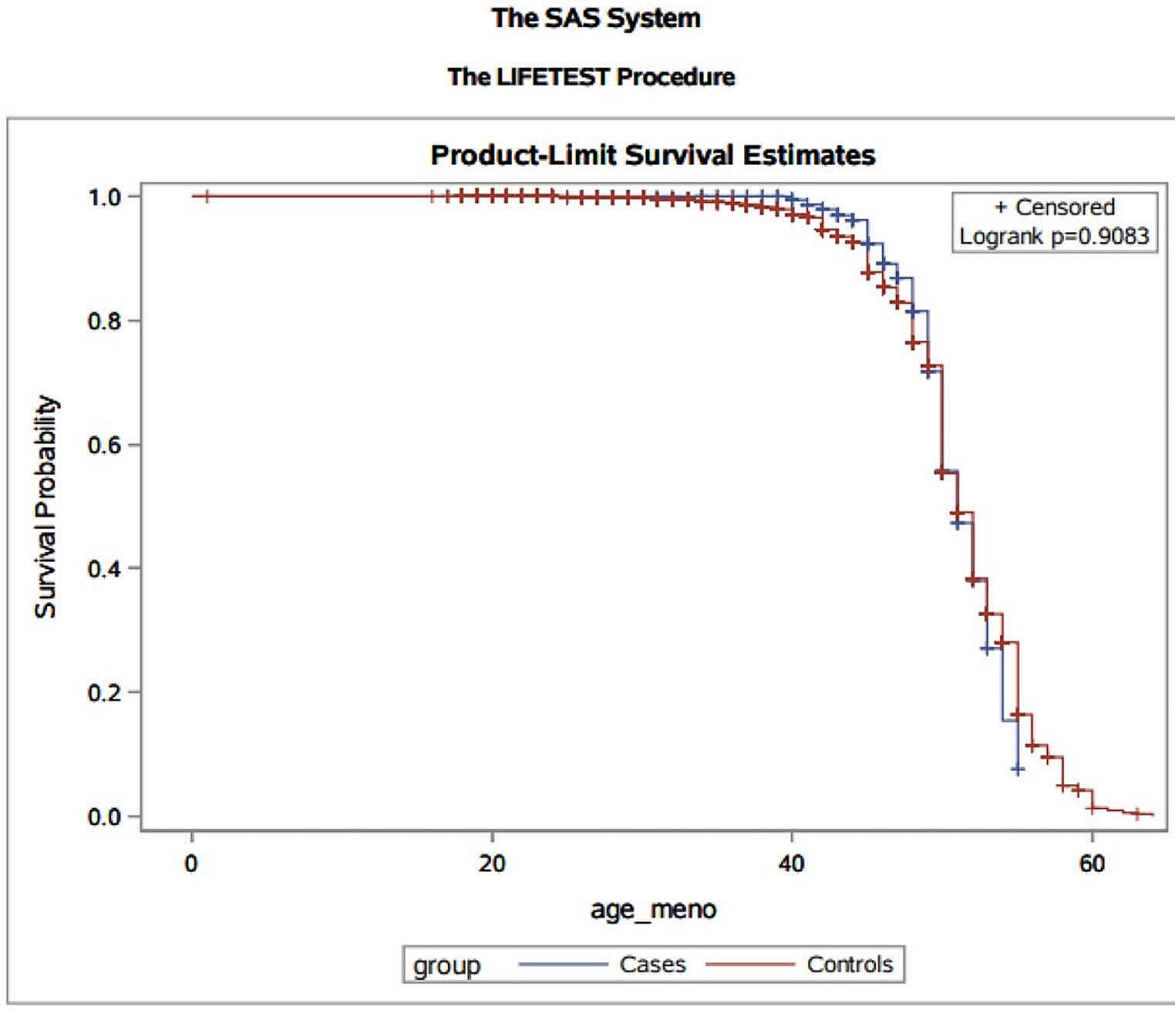
Kaplan-Meier estimates of survival curves for age at menopause in cases and controls

We compared the age of menopause of the 167 patients to a control cohort from the general population. Patients were considered informative until the timepoint of any medical event possibly affecting fertility (i.e., chemotherapy, ovariectomy, and / or hysterectomy). There was no difference in the age at menopause between the LS patients and the control cohort (*p* = 0.9083) (Fig. 1). This was also true, when the carriers of *EPCAM* deletions were excluded (*p* = 0.93). As an important role in menopause is especially described for MLH1, carriers of pathogenic *MLH1* variants (77 patients) were compared to the rest of the LS cohort. Again, there was no significant difference (*p* = 0.15). Carriers of pathogenic *MLH1* variants even seemed to have a slightly higher age at menopause.

Nearly﻿ half of our patients (75 patients, 44.9%) had already had at least one cancer before the age of 45 years. Noteworthy, one third of the patients (58 women, 34.7%) had events (mostly medical procedures) possibly affecting fertility before the age of 45 years, therefore affecting the fertility of these patients in the childbearing time. Regarding the first event, possibly affecting fertility, 39 of these patients (67.2%) had been diagnosed with cancer (mainly colorectal and endometrial cancer, two ovarian cancers and two cervical cancers), 20 of these also required chemotherapy at that timepoint. In the context of cancer therapy, 13 women underwent HE because of cervical, endometrial, or ovarian cancer; in 3 patients HE was conducted in the context of colorectal and breast cancer surgery; 3 women opted for prophylactic HE at the timepoint of cancer diagnosis.

In the other 19 women (32.8%), not cancer-related events occurred before the age of 45 years. In 11 patients (19%), HE was necessary due to other medical reasons (e.g. hypermenorrhea, myoma); three patients underwent prophylactic HE because of the high cancer risk for LS patients. Five patients (8.6%) developed menopause under the age of 45 without any obvious iatrogenic influences; but of note, one of these women had undergone abdominal surgery for colorectal cancer at the age of 24 years (20 years before menopause). Age-dependent iatrogenic factors affecting fertility are summarized in Table 2.

**Table 2:** Age-dependent iatrogenic factors affecting fertility

Patient age	Hysterectomy, all	Prophylactic hysterectomy	Hysterectomy in the context of cancer surgery	Hysterectomy due to other (gynaecological) reasons	Chemotherapy	Menopause	Menopause without iatrogenic influence
< 35 y	3 (1.8%)	0 (0%)	2 (1.2%)	1 (0.6%)	5 (3%)	4 (2.4%)	0 (0%)
< 40 y	10 (6%)	0 (0%)	6 (3.6%)	4 (2.4%)	14 (8.4%)	8 (4.8%)	0 (0%)
< 45 y	37 (22.2%)	7 (4.2%)	16 (9.6%)	14 (8.4%)	26 (15.6%)	23 (13.8%)	5 (3%)
< 50 y	67 (40.1%)	15 (9%)	32(19.2%)	20 (12%)	37 (22.2%)	72 (43.1%)	24 (14.4%)
all	92 (55.1%)	19 (11.4%)	51 (30.5%)	22 (13.2%)	48 (28.74%)	122 (73.1%)	50 (29.9%)

To explore influences on fertility in our patients, we compared the number of children from the patients to the general population. In our patient cohort (median birth year 1960), each patient had on average 1.9 children, the average age at birth of the first child was 26 years. This is in concordance to the general population: according to the federal office of statistics, the maternal age at the birth of the first child for women in the birth years 1959–1962 was between 25 and 26 years, the average number of children was 1.7 in this cohort.

## Discussion

Eight of the LS probands reported menopause before the age of 40 years, therefore fulfilling the diagnostic criteria for POF. As in all patients premature menopause occurred in the context of chemotherapy or premenopausal HE and ovariectomy, our data do not indicate an elevated risk for POF in a cohort of 167 Lynch syndrome patients.

Statistical comparison of the age at menopause of the informative LS patients to a cohort taken from the general population revealed no differences between the cohorts. This was also true if carriers of pathogenic *EPCAM* deletions were excluded, as *EPCAM* is not an MMR gene itself. Carriers of *MLH1* pathogenic variants (78 of 167 patients) even tended to a higher age at menopause compared to the rest of the group.

In mice, infertility has been shown for knockout mice of MMR genes not carrying a functional gene copy. Male knockout mice for *Mlh1* are sterile and show abnormalities in meiotic synapsis. Similar results were found for *Mlh3* and *Msh5*. Female MMR knockout mice are unable to produce oocytes and are infertile [2, 6, 7]. Therefore, it is likely, that one functional copy of the MMR genes (as it is present in heterozygous LS carriers) is sufficient to ensure normal meiosis and fertility. This would be in line with our results not indicating a reduced age of menopause in LS. Patients with pathogenic variants affecting both copies of one MMR gene have an even higher cancer risk and different tumor spectrum compared to LS patients, a condition called constitutional MMR deficiency (CMMRD). At the moment, little is known regarding fertility in these patients and data will be difficult to obtain, as CMMRD patients are rare, have a high rate of early mortality, and are often treated for cancer before considering family planning. The different genetic causes (different genes, combinations of loss of function variants with hypomorphic variants) also might affect fertility differently.

Studies in mice models showed however, that heterozygous missense variants in *Mlh1* can have different effects on meiosis, depending on their location within the gene and within certain domains. MMR variants that do not lead to nonsense mediated decay of the respective RNA but to the expression of functionally impaired MMR proteins might affect meiosis via a dominant negative effect. Therefore, it is possible, that some of the LS patients might still have an impaired fertility. Interestingly, 6 out of 7 patients with *MLH1* missense variants were younger than 50 years at menopause, but only two patients younger than 40 years (one receiving chemotherapy and one with HE and ovariectomy). Larger patient cohorts will be required for further investigation. However, recruiting these cohorts might be challenging, given that many patients opt for prophylactic HE after childbearing making determination of age at menopause difficult.

So far, in humans POF has only been described for carriers of other MMR genes not causative for LS. The group of Mandon-Pépin et al. looked for pathogenic germline variants in four genes involved in meiosis in a group of 41 women with unexplained POF. Two of these patients harbored heterozygous germline variants in the MMR gene *MSH5* [8]. Both patients were found to have POF after initially normal ovarian function. These results are in line with mice experiments, showing that initially normal functioning ovaries of *Msh5* knockout mice show rapid degeneration leading to infertility [7]. Future studies will show, if there is a connection between POF and MMR genes unrelated to LS.

Additionally, many of our patients (66.5%) have already had cancer and thus, also cancer-related treatment before menopause. This is not unusual for LS patients, as for *MLH1* and *MSH2* the risk for any cancer until the age of 50 years is 38% and 42% according to data from the prospective LS database [3]. As expected, colorectal and endometrial cancer were the most common entities. Additionally, our patients have been diagnosed with a variety of other cancers, mainly from the LS spectrum. As our cohort included many index cases (85%), cancer risks might be overrated. However, the high risks show nevertheless that premenopausal cancer diagnoses are common among LS patients and might very well affect family planning in these patients.

Cancer treatment can directly affect fertility as well as age at menopause. This is not only true for gynaecological cancers, but also for other entities like colorectal cancer, as postoperative adhesions might affect tubo-ovarian function as well. In the context of our findings with a high rate of events and medical procedures influencing fertility in our patient cohort, it would be expected that our patients would have had less children compared to women of the general population at the same age. Interestingly, our data do not support this hypothesis. The median birth year of our patients was 1960. On average, the women in our cohort got 1.9 children each, which is in concordance with the average number of children in the general population for their birth cohorts. Childbearing age (on average 26 years at first child) was also in accordance with the general German population for women born between 1959 and 1962 [9]. Our patients therefore usually started childbearing before any of the above-mentioned events reducing fertility took place. Therefore, it is comprehensible, why these events did not statistically affect family planning in our patient cohort. But nowadays, as the age at first child is considerably higher (30,2 years on average for the German population in 2020), medical events in LS patients more affect the main childbearing time for these patients. Therefore, LS patients should be aware of this possible limitation in family planning.

In conclusion, we did not find a higher risk for POF or an earlier age of menopause in women with LS. However, due to the high premenopausal cancer risk associated with cancer-related treatment, LS patients are nevertheless facing possible limitations regarding fertility (i.e., shortening the time for childbearing), in particular since the average childbearing age has increased since 1960. These issues and methods of artificial reproduction, if applicable, should be considered in family planning and therefore discussed with those patients in childbearing age.

## Data Availability

No datasets were generated or analysed during the current study.
